# Pancreatic Beta Cell Identity in Humans and the Role of Type 2 Diabetes

**DOI:** 10.3389/fcell.2017.00055

**Published:** 2017-05-23

**Authors:** Piero Marchetti, Marco Bugliani, Vincenzo De Tata, Mara Suleiman, Lorella Marselli

**Affiliations:** ^1^Department of Clinical and Experimental Medicine, University of PisaPisa, Italy; ^2^Department of Translational Medicine, University of PisaPisa, Italy

**Keywords:** beta cell, diabetes, transcription factors, insulin secretion, beta cell ultrastructure

## Abstract

Pancreatic beta cells uniquely synthetize, store, and release insulin. Specific molecular, functional as well as ultrastructural traits characterize their insulin secretion properties and survival phentoype. In this review we focus on human islet/beta cells, and describe the changes that occur in type 2 diabetes and could play roles in the disease as well as represent possible targets for therapeutical interventions. These include transcription factors, molecules involved in glucose metabolism and insulin granule handling. Quantitative and qualitative insulin release patterns and their changes in type 2 diabetes are also associated with ultrastructural features involving the insulin granules, the mitochondria, and the endoplasmic reticulum.

## Introduction

The pancreatic beta cells are endocrine cells that synthetize, store, and release insulin, the anti-hyperglycemic hormone that antagonizes glucagon, growth hormone, glucocorticosteroids, epinephrine, and other hyperglycemic hormones, to maintain circulating glucose concentrations within a narrow physiologic range. Beta cells have an average diameter of 10 μm, contain about 20 pg insulin per cell, and are the predominant cell type in the pancreatic islets (50–80% of all islet endocrine cells) (Marchetti and Ferrannini, 2015). In the human pancreas, beta cell mass has been reported to vary from 0.6 to 2.1 g, and the amount of insulin in the gland has been observed to range from 50 to 250 ug/g (Marchetti and Ferrannini, 2015). In an adult human being, beta cells release ~30–70 U insulin per day (mainly depending on body weight), half of which is secreted after meals and the rest under basal conditions. The release of this hormone is regulated by a complex network of many different triggering, potentiating or inhibiting signals, which allows the supply of the hormone in amount, kinetics and adaptability to match the minute-by-minute variable needs of the body (Henquin, 2005). In this article we will describe some key distinguishing molecular, functional, and ultrastructural features of the human beta cells, with emphasis on changes occuring in the case of type 2 diabetes.

## Molecular identity

Insulin expressing beta cells are the first endocrine cells to appear in the human pancreas, at 7.5–8 weeks of gestation, i.e., approximately 3 weeks after the initial formation of the dorsal and ventral pancreatic buds (Pan and Brissova, 2014). Shortly after, glucagon and somatostatin-expressing cells appear (gestational week 8), followed by ghrelin-expressing cells (gestational week 9; Piper et al., 2004; Pan and Brissova, 2014). However, a few authors have reported that beta cells are detectable from gestational week 9 onward, whereas glucagon expressing cells appear already at week 8 (Meier et al., 2010). In addition, during the early fetal period, small proportions of cells may co-express both insulin and glucagon (Meier et al., 2010; Riedel et al., 2012).

The transcription factor NGN3 is needed to commit progenitor cells toward an endocrine cell phenotype (Gradwohl et al., 2000; Pan and Brissova, 2014). Expression of NGN3 peaks by the end of the first trimester and disappears at about the 35th week of gestation. In the early (8th–11th) weeks of gestation of the human fetal pancreas, cells co-expressing NGN3 and PDX1, NGN3 and insulin, NGN3 and glucagon have been observed (Lyttle et al., 2008). Additional transcriptional regulators specifically associated with pancreatic endocrine cells at these stages include PAX6, NKX2.2, NKX6.1, ISLET1, and PAX4 (Lyttle et al., 2008; Sarkar et al., 2008; Jennings et al., 2015). Also in the adult human pancreas, maintenance of beta cell identity is associated with the presence of key transcription factors (in particular PDX1, FOXO1, MAFA, NKX6.1), and changes in their expression and/or localization have been described in the islets from type 2 diabetic (T2D) individuals, contributing to beta cell de-differentiation (i.e., the regression to a progenitor-like state) in this disease (Talchai et al., 2012; Guo et al., 2013; Kitamura, 2013; Rutter et al., 2015; Spijker et al., 2015; Cinti et al., 2016; Remedi and Emfinger, 2016). In particular, a study (Cinti et al., 2016) reported the use of a de-differentiation score based on markers of endocrine lineage, beta cell specific transcription factors, and the endocrine progenitor cell marker, aldehyde dehydrogenase 1A3, to show a three- to four-fold increase of de-differentiated cells in islets from type 2 diabetes. On the same line, other authors (Spijker et al., 2015) have found a several fold higher frequency of cells double positive for insulin and glucagon in the diabetic samples, together with a 5 times increase of cells that were Nkx6.1 and glucagon positive, but insulin negative (a finding that was correlated with the presence and extent of islet amyloidosis).

In addition to transcription factors, the expression of several additional genes has been found to change in T2D islets by real-time RT-PCR, compared to adult non-diabetic (ND) samples. For instance, GLUT2 and glucokinase were found to be downregulated in type 2 diabetic islets (Del Guerra et al., 2005). Furthermore, changes of the expression of genes involved in regulating cell redox balance have been shown (Marchetti et al., 2004): NADPH oxidase was found to be increased and that of Mn and Cu/Zn superoxide dismutases decreased in diabetic islets, together with enhanced expression of catalase and GSH peroxidase.

More comprehensive assessments have been performed by studying the transcriptomic features of isolated islets by microarray o RNA-sequencing (see also Table 1). Gunton et al. first profiled the gene expression of islets from 7 ND and 5 T2D individuals using oligonucletotide microarrays (Gunton et al., 2005). Significant (*p* < 0.01) changes in the expression of 370 out of the 44,298 genes and ESTs examined were found, with downregulation of several genes relevant for beta cell function, such as HNF4, but also for glucose-sensing (including GLUT2) and insulin-signaling. Afterwards, independent analysis of 4 ND and 4 T2D islet preparations showed reduced expression in T2D islets of factors implicated in mitochondrial function and regulated exocytosis (Ostenson et al., 2006; MacDonald et al., 2009). More recent work with 7 ND and 6 T2D islet preparations pointed to the occurrence of changes in genes of the ubiquitin–proteasome system (Bugliani et al., 2013), with however several additional alterations in genes and pathways affecting many islet cell structures and functions, from glucose metabolism and insulin granule exocytosis to oxidative stress and cell turn-over. Groop and his collaborators have largely contributed to this field (Mahdi et al., 2012; Taneera et al., 2012, 2015; Fadista et al., 2014). They originally studied islets from 54 ND to 9 T2D organ donors (Taneera et al., 2012), with the addition of some more cases in experiments thereafter. By the use of several integrated biosystems analysis approaches, they identified genes such as CHL1, LRFN2, RASGRP1, PPM1K, PPP1R1A, FAM105A, ENO2, PLCDX3, GNG5, TSPAN33, TMED6, and PAK7 to be related to the disease associated reduction of insulin secretion (Taneera et al., 2012; Fadista et al., 2014). In addition, they reported that the expression of SRFP4, which encodes secreted frizzled-related protein 4, is upregulated in T2D islets, possibly representing a link with beta cell inflammation (Mahdi et al., 2012). More lately, preliminary data have been presented (Solimena et al., 2016; EASD annual meeting) obtained with islets isolated either by enzymatic digestion from 103 organ donors (84 ND and 19 T2D), or by laser capture microdissection from surgical specimens of pancreatectomized patients (including 32 ND and 36 T2D individuals). Comparative transcriptomic analysis identified 19 genes differentially expressed (FDR ≤ 0.05, fold change ≥ 1.5) in T2D as compared to ND islets, including GLUT2, ARG2, PPA1R1A; in addition, systems biology approaches identified HNF1A, PDX1, and REST as drivers of gene co-expression modules correlated with impaired insulin secretion, that were enriched in 14 out of the 19 differentially expressed genes.

**Table 1:** **Some studies reporting transcriptomic data in type 2 diabetic (T2D) and non-diabetic (ND) islets or beta cells**.

**Technique**	**T2D vs. ND**	**References**
Islet Microarray	Dysregulation of 370 genes/EST	Gunton et al., 2005
	Focus on genes involved in exocytosis	Ostenson et al., 2006
	Focus on genes involved in mitochondrial function	MacDonald et al., 2009
	Focus on genes of the ubiquitin-proteasome system	Bugliani et al., 2013
	Global map of genes associated with beta cell dysfunction	Taneera et al., 2012
	Focus on Secreted frizzle-related protein 4	Mahdi et al., 2012
Islet RNA seq	Global genomic and transcriptomic analysis	Fadista et al., 2014
Laser-capture microdissected beta cells	Almost 2,000 transcripts dysregulated	Marselli et al., 2010
Single cell RNA seq	48 transcripts dysregulated	Xin et al., 2016
	75 transcripts dysregulated	Segerstolpe et al., 2016

However, islets represent an heterogeneous combination of different endocrine cells, and efforts are being made to identify specific beta cell features in ND and T2D humans. By using a technique based on laser capture microdissection, Marselli et al. studied beta cells from 10 ND to 10 T2D organ donors (Marselli et al., 2010). Several thresholds were employed to assess differences: with the lower confidence bound (LCB) parameter at the cutoff of 1.2, 2,062 differentially expressed probe sets, corresponding to 1,920 transcripts, were identified (1,203 were upregulated and 717 downregulated in T2D islets); with the *p*-value set at <0.05, 7,164 probe sets, corresponding to 6,384 transcripts, were found to be dysregulated in T2D islets (3,464 upregulated and 2,920 downregulated); with a more stringent *p*-value (<0.01), 2,133 probe sets, corresponding to 1,870 transcripts were different in the diabetic samples (1,006 upregulated and 864 downregulated). Among the differentially expressed genes, there were some linked to glucotoxicity, oxidative stress, cell cycle, apoptosis, endoplasmic reticulum stress, and pancreatic regeneration. In addition, the expression of a proportion of the genes found in GWAS studies to be associated with T2D was observed to change in T2D islets, including IGF2BP2, TSPAN8, and HNF1B (upregulated), and JAZF1 and SLC30A8 (downregulated). HNF1A was also downregulated, whereas PAX6 and PDX1 (that showed however a low signal strength) were upregulated in the diabetic samples. Transcription factors that did not change included NKX2-2, NKX6.1, NEUROD1, PAX4, HNF4A, NGN3, and MAFB.

More recently, RNA-seq studies have been published using single human pancreatic cells, obtained from islets isolated from ND and T2D organ donors by enzymatic digestion (Xin et al., 2016) or dissociation and FACS sorting (Segerstolpe et al., 2016). The comparison of beta cells isolated from ND (respectively from 12 and 6 preparations) or T2D (respectively from 6 and 4 preparations) showed that 48 (Xin et al., 2016) or 75 (Segerstolpe et al., 2016) transcripts were differentially expressed between control and diabetic samples. Of note, only one gene (FXYD2) was regulated in both studies, but in opposite directions. It is conceivable that the use of single cell RNA seq techniques will have a rapid development in the future, including the possibility of its use to better define cell type diversity and the dynamics of cell development, and also provide quantitative and comprehensive information (Kumar et al., 2017).

Therefore, in essence the beta cell is characterized, at the molecular level, by expressing insulin since its early embryogenesis, and a few transcription factors are key to its maturation and maintenance of competence. Many molecules are implicated in beta cell function and turn-over, and several have been found to change in T2D islets and/or beta cells. However, due to islet and beta cell heterogeneity (differences between donors and portions of the pancreas, diverse distribution of the islets in the pancreatic lobules, variable islet sizes, unpredictable proportions, and location of endocrine cells in the islets; Bonner-Weir and Aguayo-Mazzucato, 2016; Roscioni et al., 2016; Gutierrez et al., 2017), as well as to limitations of the available techniques, a precise molecular profiling of the “typical” T2D beta cell is still missing.

## Functional identity

The key role of the beta cells is to produce and secrete insulin in a tightly regulated manner, to maintain circulating glucose concentrations in the (narrow) physiological range (Cavaghan and Polonsky, 2005; Henquin, 2005; Poitout et al., 2015). Of the several physiological compounds known to stimulate insulin secretion (Table 2), glucose is by far the most important. The increase of insulin release in response to oral and intravenous glucose is dose-dependent and the secretion is greater after oral than intravenous administration. This phenomenon has been named incretin effect (Nauck et al., 1986), and appears to be due to the release of gastrointestinal hormones and other mechanisms elicited by glucose in the enteric tract. The secretion of insulin by the beta cell is dynamic. Intravenous glucose infusion determines a biphasic insulin response, consisting of an early rapid peak (first phase, lasting a few minutes), followed by a slower and more prolonged increase (second phase). In addition, insulin secretion is pulsatile (Cavaghan and Polonsky, 2005). The pulses period is normally of 8–10 min and they superimpose on longer fluctuations. The islets themselves are probably the “controllers” of the high-frequency pulses, whereas the control of the lower-frequency oscillations may be from outside the islets.

**Table 2:** **Main physiological regulators of insulin secretion**.

Nutrients	Glucose (Henquin, 2005)
	Amino acids (Newsholme et al., 2014)
	Free fatty acids (>C12) (Warnotte et al., 1999; Morgan and Dhayal, 2009)
	Ketones (Panten et al., 2013)
Hormones	GLP-1 (Drucker and Nauck, 2006)
	GIP (Drucker and Nauck, 2006)
	CCK (Ning et al., 2015)
	Glucagon (Unger, 1985)
	VIP (Sanlioglu et al., 2012)
	Gastrin (Rehfeld, 2011)
	Secretin (Glaser et al., 1988)
	PACAP (Sanlioglu et al., 2012)
Neurotransmitters	Acetylcholine (Gilon and Henquin, 2001)
	Norepinephrine (Straub and Sharp, 2012)

**Figure 1:**
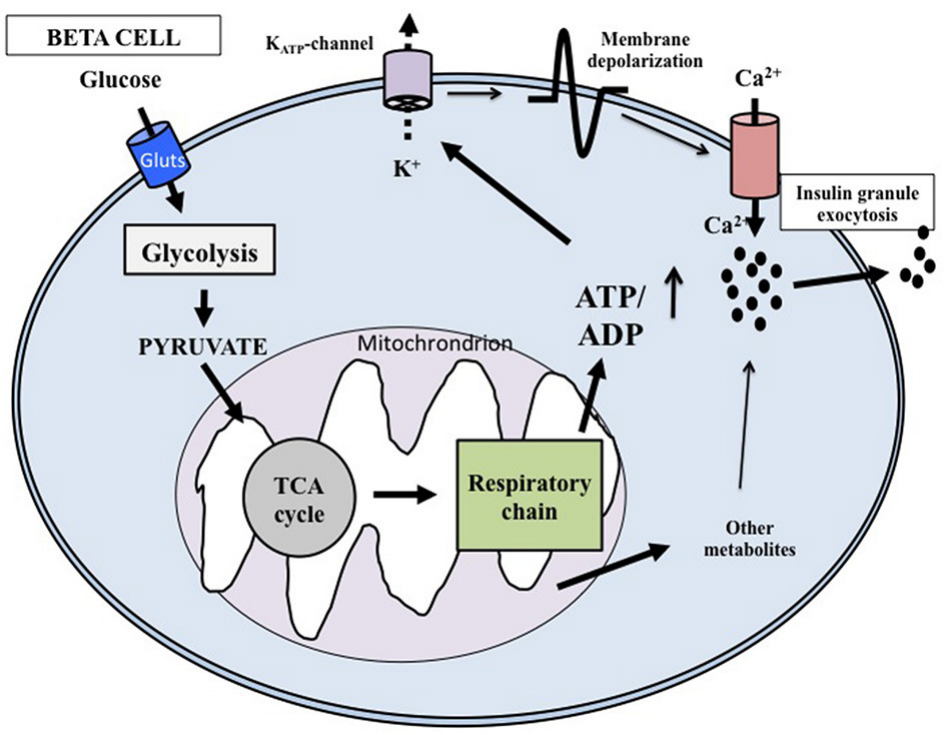
**Key mechanisms of glucose-induced insulin secretion**. Glucose enters the beta cell rapidly through specific glucotransporters and is phosphorylated by glucokinase; this step regulates metabolic flux through glycolysis. After pyruvate has entered the mitochondria, reducing equivalents are produced by the tricarboxylic acid cycle. ATP is then generated and the augmented ATP/ADP ratio causes the closure of the ATP-sensitive K^+^ (K_ATP_) channels in the membrane. The successive depolarization of the plasma membrane leads to influx of extracellular Ca^2+^ and activation of exocytosis. Additional products generated in the mitochondria may also affect insulin release.

At the cellular level, glucose exerts its effects through intermingled mechanisms that overall are now defined according to models of stimulus-secretion coupling (Juhl and Hutton, 2004; Henquin, 2011). Glucose is both a trigger and an amplifier of insulin secretion. As synthetized in Figure 1 (based on: Juhl and Hutton, 2004; Cavaghan and Polonsky, 2005; Henquin, 2005, 2011; Marchetti et al., 2008; Poitout et al., 2015) its transport into the beta cell cytoplasm is facilitated by glucose transporter 1 and 2 (GLUT1 and GLUT2). Then the exose is rapidly phosphorylated by glucokinase (a high Km isoform of hexokinase) to begin the glycolytic process. Since glucose-6-phosphate is produced without allosteric inhibition, glucokinase itself is the controller of the rate of glycolysis; the enzyme is therefore considered the molecular glucose sensor that regulates insulin release from the beta cell. Glycolysis ends with the production of pyruvate, the three-carbon molecule that enters the mitochondria to be oxidated. Pyruvate is first converted by pyruvate kinase to acetyl-CoA, that in turn reacts with oxalocetate in a reaction catalyzed by citrate synthase to obtain citrate, a component of the tricarboxylic acid (TCA) cycle. Activation of this cycle leads to the generation of NADH and FADH_2_, and these reducing equivalents are employed in the production of ATP in the electron transport chain. The ATP produced in the mitochondria is then transported to the cytoplasm by the adenine nucleotide translocator (ANT). The augmented cytosolic ATP levels determine the closure of the K_ATP_ channels, reduction of K^+^ conductance and plasma membrane depolarization. Therefore, the K_ATP_ channels couple the metabolic activity of the beta cell to the electrical state. In turn, depolarization of the plasma membrane induces the opening of L-type voltage-dependent Ca^2+^ channels, causing a rapid depolarization to a plateau potential with superimposed bursts of Ca^2+^ potentials. Ca^2+^ influx into the beta cell cytoplasm mobilizes the insulin granules, followed by membrane docking and exocytosis.

As mentioned above, glucose can also amplify the secretion of insulin, which means that products of glucose metabolism potentiate the exocytosis of the insulin granules without any further increase of cytosolic Ca^2+^ concentrations (Juhl and Hutton, 2004; Henquin, 2005, 2011). The mechanisms responsible of the amplifying pathway are not clearly defined yet, but possible mediators include metabolites produced in the mitochondria during glucose metabolism.

In addition to glucose, several other compounds affect insulin secretion from the beta cell (Table 2). A few aminoacids, either alone or in combination, induce or potentiate the release of the hormone by metabolic mechanisms, biophysical processes, or both (Newsholme et al., 2014). Some of them (such as leucine and glutamate) are metabolized in the mitochondria, thus fueling the TCA cycle. Arginine, instead, depolarizes the plasma membrane after it is transported into the cytoplasms, due to its positive charges. This depolarization activates the voltage-dependent Ca^2+^ channels which in turn permits the entry of this ion, leading to the successive insulin granule exocytosis. Free fatty acids can stimulate insulin release by either acting as metabolic substrates (modulating the oxidation of fuel molecules in the mitochondria) and/or by binding to specific G-protein coupled receptors (GPRs, in particular GPR40 and also GPR119 and GPR120) that are expressed on the membrane of beta cells (Warnotte et al., 1999; Morgan and Dhayal, 2009). Finally, the GPRs system is also involved in the modulation of insulin release induced by hormones and neurotransmitters. In this regard, the role of the incretin hormone GLP-1 has been specifically and extensively investigated, mainly in consideration that the GLP-1 system has become a target for the treatment of type 2 diabetes (Drucker and Nauck, 2006). GLP-1 is secreted from the enteroendocrine L-cells after nutrient ingestion and potentiate insulin release glucose dependently. GLP-1 mechanisms of action on insulin secreting cells include activation of adenylate cyclase as well as production of IP-3 and DAG. The consequent activation of PKA-dependent and independent processes, leads, mainly through Epac2 action and the PKA-mediated phosphorylation of several substrates, to increased insulin granule movement and ion channels activity (Doyle and Egan, 2007). Additional regulators of insulin-release are reported in Table 2 (Gilon and Henquin, 2001; Glaser et al., 1988; Newsholme et al., 2014; Ning et al., 2015; Panten et al., 2013; Rehfeld, 2011; Sanlioglu et al., 2012; Straub and Sharp, 2012; Unger, 1985).

In type 2 diabetes there are several quantitative and qualitative alterations of insulin secretion (Cavaghan and Polonsky, 2005; Henquin, 2005; Marchetti and Ferrannini, 2015). Usually reported changes include blunted or absent first phase insulin release to intravenous glucose, delayed and/or reduced responses to the ingestion of a mixed meal, and over the years, decreased second phase release and secretion to non-glucose stimuli. In addition, alterations of pulsatile patterns and increased proinsulin release have been described. Studies with isolated human islets have allowed direct characterization of some features of insulin release in T2D *ex-vivo*. In early experiments it was shown that glucose-induced insulin release was decreased with T2D islets, whereas the secretory response to combined L-leucine and L-glutamine stimulation was less markedly impaired (Fernandez-Alvarez et al., 1994). In another study, islets prepared from 9 ND and 8 T2D donors were evaluated *ex-vivo* by the perifusion technique (Deng et al., 2004). The Authors found that the diabetic islets secreted lower amount of insulin upon stimulation with increasing glucose concentrations and also showed a higher threshold needed to initiate insulin release. These findings were essentially confirmed in an independent article from a different group (Marchetti et al., 2004). Lately, insulin secretion was measured in ND and T2D islets upon challenge with glucose, glibenclamide and arginine (Del Guerra et al., 2005). In this study, the diabetic islets released significantly less insulin than control islets in response to glucose and also during glibenclamide and arginine stimulation; however, they secreted more insulin in response to non-glucose stimuli than in response to the exose, suggesting more marked beta cell defects when glucose is the secretagogue compound. As mentioned earlier in the present article, the three tested secretagogues (glucose, glibenclamide, and arginine) induce insulin secretion through different mechanisms (Juhl and Hutton, 2004; Cavaghan and Polonsky, 2005; Henquin, 2005, 2011; Marchetti et al., 2008; Poitout et al., 2015): glucose needs to enter the beta cell and be metabolized to produce ATP, whereas glibenclamide causes insulin secretion by binding to the K_*ATP*_ channels and determong their closure; finally, arginine enters the beta cell through the CAT 2A cationic aminoacid transporter and induces excess of positive charges with subsequent depolarization of the plasma membrane. It is therefore possible that specific defects of glucose handling may be present in the T2D beta cell. Consistent with this, the expression of glucose transporters has been found to be lower in T2D than ND islets (Gunton et al., 2005; Taneera et al., 2012; Solimena et al., 2016; EASD annual meeting). More importantly, several evidences indicate that the mitochondria could play a major role (Marchetti et al., 2008; Mulder and Ling, 2009). In fact, the activities of the mitochondrial enzymes glycerol phosphate dehydrogenase, pyruvate carboxylase and succinyl-CoA:3-ketoacid-CoA transferase have been reported to be ~65–90% lower in the diabetic compared with control islets (MacDonald et al., 2009). Furthermore, ATP citrate lyase, a cytosolic enzyme involved in the mitochondrial citrate pyruvate shuttle, was also reduced of more than 50% (MacDonald et al., 2009). All this could explain, at least in part, the observation that glucose oxidation is reduced in T2D (Del Guerra et al., 2005). In addition, T2D beta cell mitochondria look round shaped and with lower density by electron microscopy analysis, and when adenine nucleotide content was quantified, it was observed that cells from T2D subjects did not increase their ATP content upon acute glucose stimulation (Anello et al., 2005). Hence, the ATP/ADP ratio was almost 50% lower in T2D than control islets, and, accordingly, altered hyperpolarization of the mitochondrial membrane, as well as augmented expression of UCP-2, complex I, and complex V of the respiratory chain were observed (Anello et al., 2005). On top of all this, a more general defect contributing to defective insulin secretion in T2D beta cells could be the reduced expression of molecules involved in insulin granule exocytosis, such as those of the SNARE complex and SNARE-modulating proteins (syntaxin-1A, SNAP-25, VAMP-2, Munc 18, Munc 13-1, and synaptophysin; Ostenson et al., 2006).

Finally, insulin release from T2D beta cells has been reported to show alterations of the pulsed pattern (Lin et al., 2002), and it is associated with the release of four- to five-fold higher proportions of proinsulin, indicating less efficient conversion of proinsulin to insulin (Cavaghan and Polonsky, 2005; Henquin, 2005; Marchetti and Ferrannini, 2015).

Therefore, the functional identity of the beta cell is indissolubly linked to its capacity to secrete insulin in response to increased glucose levels, which is regulated by several intracellular organelles and mechanisms and whose impairment is the hallmark of type 2 diabetes.

## Ultrastructural identity

**Figure 2:**
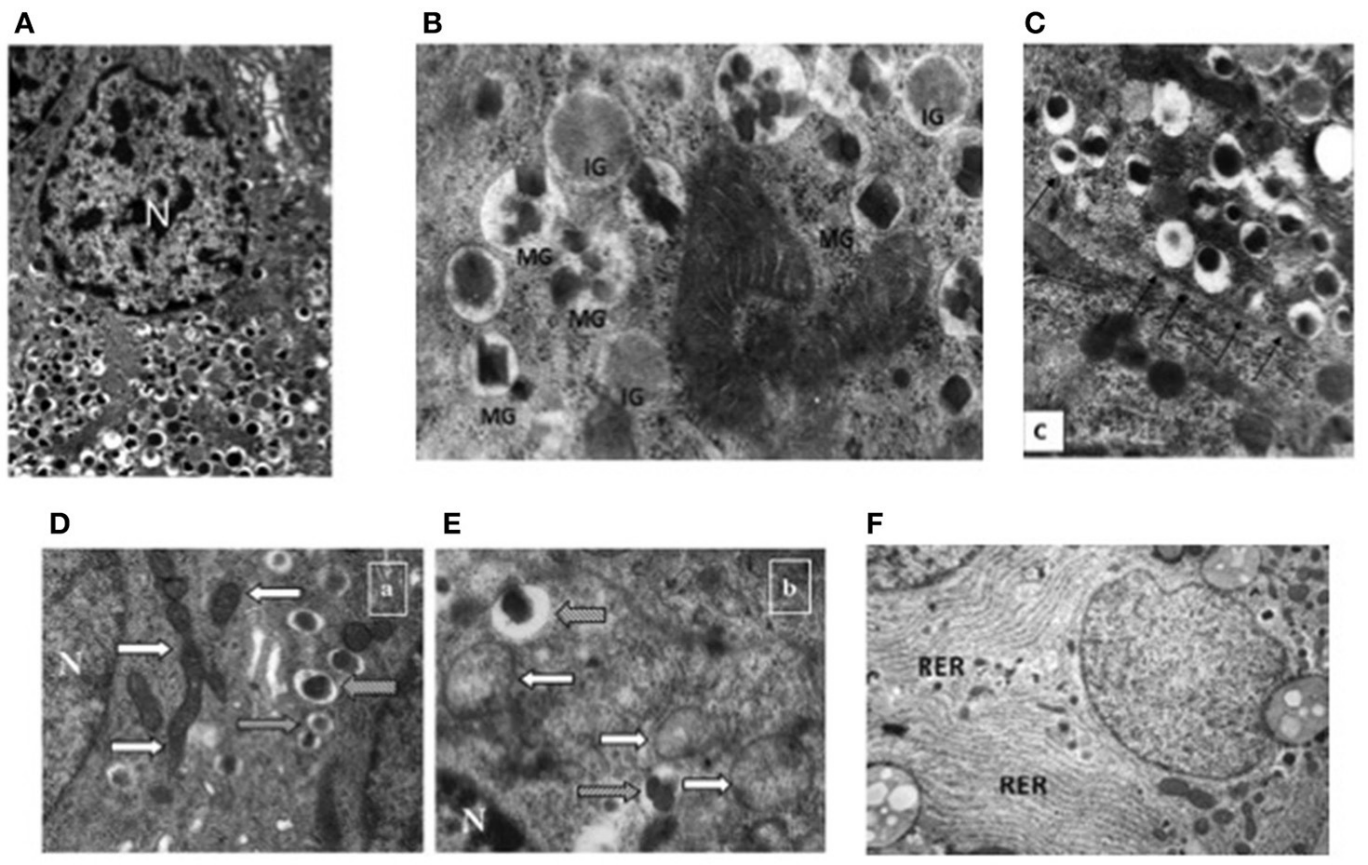
**Electron microscopy images of non-diabetic (ND) and type 2 diabetic (T2D) beta cells. (A)** Insulin granules in an ND beta cell, with their typical ultrastructure; **(B)** mature (MG) and immature (IG) insulin granule in an ND beta cell; **(C)** docked insulin granules (arrows indicate the beta cell membrane); **(D)** mitochondria (white arrows) in an ND beta cell (gray arrows indicate insulin granules); **(E)** mitochondria (white arrows) in a T2D beta cell (gray arrows indicate insulin granules); **(F)** endoplasmic reticulum (RER) in a T2D beta cell (the RER is usually barely detectable in ND beta cells—see panel **A**) N, Nucleus. Reproduced with permission from Anello et al. (2005) and Masini et al. (2012).

At the ultrastructural level, beta cells are identified by the presence of the typical insulin granules (Lacy and Hartroft, 1959; Orci, 1985; In't Veld and Marichal, 2010; Ivanova et al., 2013). Insulin granules are characterized by an electron dense core and a clear peripheral mantle (Figure 2A), their size is around 300 nm and their number has been estimated to be ~10,000 per beta cell. Depending on the level of maturation, the mantle of the granules may contain proinsulin that is not yet fully processed; when the cleavage of this precursor is unsufficient, the dense core may be absent and typical immature “gray” granules may be found (In't Veld and Marichal, 2010; Masini et al., 2012) (Figure 2B). Interestingly, a proportion of insulin granules is located in close proximity of the cell membrane (Ohara-Imaizumi et al., 2004; Masini et al., 2012), representing a population defined as “docked granules” (Figure 2C), possibly involved in the first phase of insulin release. Several changes at the level of insulin granules have been reported in T2D islet beta cells (Marchetti et al., 2004; Masini et al., 2012). In fact, the overall amount of insulin granules is lower in the T2D diabetic beta cells (Marchetti et al., 2004; Masini et al., 2012); in addition, the proportion of mature “docked” granules is also lower in ND compared to T2D beta cells (Masini et al., 2012).

Further ultrastructural changes that occur in T2D beta cells involve the mitochondria and the endoplasmic reticulum (ER) (Anello et al., 2005; Marchetti et al., 2007). As mentioned above, mitochondrial dysfunction is likely to play a major role in impaired glucose-stimulated insulin release from T2D beta cells; this is accompanied by changes in the morphology of these organelles, that look round-shaped rather than elongated, with alterations in cristae appearance, reduced electron density and augmented overall volume (Figures 2D,E; Anello et al., 2005). In turn, the ER is usually more readily visible in T2D than ND beta cells (Figure 2F), showing concentrically arranged, not dilated cisternae and strongly electron-dense ribosomes bound to the membranes (Marchetti et al., 2007); when quantified, ER volume density in T2D beta cells was found to be two-fold higher than that of control beta cells (Marchetti et al., 2007).

**Figure 3:**
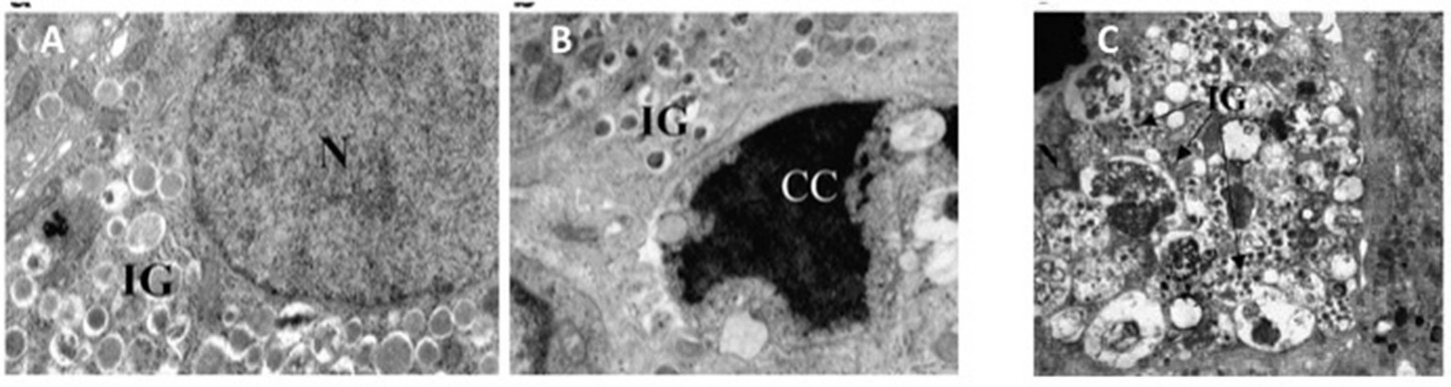
**(A)** A normal beta cell (N, nucleus; IG, insulin granules); **(B)** a beta cell with apoptotic nucleus (CC, chromatin condensation; IG, insulin granules); **(C)** a beta cell with signs of death associated with dysregulated autophagy (massive vacuole accumulation in the cytoplasm; N, nucleus; IG, insulin granules). Reproduced with permission from Marchetti et al. (2007) and Masini et al. (2009).

Although, possibly over-estimated (Marselli et al., 2014), beta cell death can contribute to the loss of beta cell functional mass in T2D (Cnop et al., 2005; Laybutt et al., 2007; Marchetti et al., 2008). In fact, the amount of apoptotic beta cells is increased in T2D islets (Cnop et al., 2005; Laybutt et al., 2007; Marchetti et al., 2008). Typically, when compared to a normal beta cell, at the EM an apoptotic beta cell show alterations such as marked chromatin condensation in the nucleus (Figures 3A,B), membrane bledding and apoptotic bodies (Galluzzi et al., 2009). However, other mechanisms may lead to beta cell death, including those associated with dysregulated autophagy (Masini et al., 2009; Watada and Fujitani, 2015): in this case, the beta cells show massive accumulation of vacuoles characterized by the presence of close double membranes surrounding organelles and/or cytoplasmic portions, in the absence of nuclear abnormalities (Figure 3C).

In summary, the T2D diabetic beta cells show several ultrastructural alterations in different intracellular compartments; although not fully pathognomonic of the diabetic condition, these changes allow inferences on the organelles and mechanisms involved in beta cell functional and survival defects.

## Conclusions

Pancreatic beta cells uniquely synthetize, store, and release insulin. Specific molecular, functional and ultrastructural features characterize these cells and their insulin secretion activity as well as survival properties. Several changes occur in type 2 diabetic beta cells, playing roles in the disease and representing possible targets for therapeutical interventions. However, the pathophysiological interplay between many different mechanisms and pathways does not allow, presently, to identify single culprits associated with the presence of type 2 diabetes. This complex scenario needs further and, at the same time, more comprehensive and fine approaches to better describe the trajectory of beta cell phenotype in the onset and progression of type 2 diabetes.

## Author contributions

All authors listed, have made substantial, direct and intellectual contribution to the work, and approved it for publication.

## Funding

European Union's Horizon 2020 Research and Innovation Programme, project T2DSystems, under Grant Agreement No. 667191.

### Conflict of interest statement

The authors declare that the research was conducted in the absence of any commercial or financial relationships that could be construed as a potential conflict of interest. The reviewer GDSX and handling Editor declared their shared affiliation, and the handling Editor states that the process met the standards of a fair and objective review.
